# Identification of *CkSNAP33*, a gene encoding synaptosomal-associated protein from *Cynanchum komarovii*, that enhances Arabidopsis resistance to *Verticillium dahliae*

**DOI:** 10.1371/journal.pone.0178101

**Published:** 2017-06-02

**Authors:** Ping Wang, Xueyan Zhang, Xiaowen Ma, Yun Sun, Nana Liu, Fuguang Li, Yuxia Hou

**Affiliations:** 1 College of Science, China Agricultural University, Beijing, China; 2 State Key Laboratory of Cotton Biology, Institute of Cotton Research of the Chinese Academy of Agricultural Sciences, Anyang, China; Nanjing Agricultural University, CHINA

## Abstract

SNARE proteins are essential to vesicle trafficking and membrane fusion in eukaryotic cells. In addition, the SNARE-mediated secretory pathway can deliver diverse defense products to infection sites during exocytosis-associated immune responses in plants. In this study, a novel gene (*CkSNAP33*) encoding a synaptosomal-associated protein was isolated from *Cynanchum komarovii* and characterized. CkSNAP33 contains Qb- and Qc-SNARE domains in the N- and C-terminal regions, respectively, and shares high sequence identity with AtSNAP33 from *Arabidopsis*. *CkSNAP33* expression was induced by H_2_O_2_, salicylic acid (SA), *Verticillium dahliae*, and wounding. Arabidopsis lines overexpressing CkSNAP33 had longer primary roots and larger seedlings than the wild type (WT). Transgenic Arabidopsis lines showed significantly enhanced resistance to *V*. *dahliae*, and displayed reductions in disease index and fungal biomass, and also showed elevated expression of *PR1* and *PR5*. The leaves of transgenic plants infected with *V*. *dahliae* showed strong callose deposition and cell death that hindered the penetration and spread of the fungus at the infection site. Taken together, these results suggest that *CkSNAP33* is involved in the defense response against *V*. *dahliae* and enhanced disease resistance in Arabidopsis.

## Introduction

Soluble *N*-ethylmaleimide-sensitive factor adaptor protein receptor (SNARE) proteins were first identified in the late 1980s and have been characterized as key components for vesicle trafficking and membrane fusion in eukaryotic cells [1, 2]. These proteins contain an evolutionarily conserved domain that comprises a characteristic sequence of 60–70 amino acids [2]. Selective membrane fusion is accomplished by the interaction between SNAREs localized in the target membranes (t-SNAREs) and those anchored to the transport vesicle (v-SNAREs) [3]. These SNAREs form a SNARE complex containing a four-helix bundle comprising of the helices Qa, Qb, Qc, and R-SNARE domains, which provide the specificity and energy required for membrane fusion [3–5]. In terms of the essential role in vesicle trafficking, SNAREs are known to be involved in key functions in plants such as promoting the formation of the cell wall [1, 6, 7], interaction with ion channel-related proteins [8–10], response to abiotic stress [11–13], and participation in plant defense against pathogens [11–16].

The synaptosomal-associated protein 25 (SNAP25) class was first described in the mammalian neuron [17]. SNAP25 homologs contain two helices that are located at the N- and C-terminal regions of the SNARE complex in the mammalian neuronal synaptic membrane [17]. AtSNAP33 was the first characterized t-SNARE SNAP25 protein in plant and is involved in diverse membrane fusion processes, including cell plate formation in cytokinesis [1]. SNAP25-type proteins are essential for the growth and development of organisms; for example, loss of functional HsSNAP29, a human SNAP25-type protein, impairs endocytic recycling and cell motility, resulting in CEDNIK syndrome (cerebral dysgenesis, neuropathy, ichthyosis and palmoplantar keratoderma) [18]. Plants with loss of functional SNAP25-type proteins gradually develop large necrotic lesions on leaves and eventually result in a lethal dwarf phenotype [1, 19]. In addition, several studies have demonstrated the involvement of SNAP25-type proteins in plant defense responses. For example, *OsSNAP32*, a SNAP25-type protein in rice, is involved the responses to abiotic and biotic stresses [20] and overexpression of *OsSNAP32* increases rice resistance to blast [15]. Expression of *AtSNAP33* increases response to pathogenic infection and mechanical stimulation [21]. HvSNAP34, a SNAP25-type protein in barley, can interact with ROR2 to mediate disease resistance against powdery mildew in the plant cell wall [22]. Moreover, the ternary PEN1-SNAP33-VAMP721/722 SNARE complex is a default secretory pathway for plant immune responses [23, 24].

*Cynanchum komarovii* Al Iljinski is a desert plant that has been used as an herbal medicine to cure fever and cholecystitis in humans. We have previously reported that protein extracts of *C*. *komarovii* seeds possess strong antifungal activity [25–27]. Transformation of Arabidopsis with *CkTLP* and *CkChn134* resulted in plant that showed resistance to the pathogenic fungus *Verticillium dahliae* [26]. This antifungal property stimulated the present study in which we sought to determine whether a gene encoding a SNAP25-type protein from *C*. *komarovii* could be used to improve crop resistance to *V*. *dahliae* via a transgenic engineering strategy.

In this study, we cloned and characterized *CkSNAP33*, a gene encoding a t-SNARE SNAP25-type protein, from *C*. *komarovii* and determined its expression at the mRNA level. We found that *CkSNAP33* transgenic Arabidopsis lines were larger in size than wild type (WT) plants and that overexpression of *CkSNAP33* enhanced Arabidopsis resistance to *V*. *dahliae*. The possible defense response was investigated by histological analyses of cell death and callose deposition.

## Material and methods

### Plant and fungal growth conditions and treatments

*C*. *komarovii* was grown in a mixture of soil and vermiculite (2:1, w/w), in a greenhouse at 16–28°C under a 16/8-h photoperiod. Arabidopsis seeds (Columbia ecotype) were sterilized with 75% ethanol and 4% NaClO solution and then sowed onto Murashige-Skoog (MS) plates (1×MS salts, 1×MS vitamins, 2% sucrose, 0.8% agar, pH 5.7). After vernalization for 3 days at 4°C, the plates were incubated in chamber under 16-h light (22°C) /8-h dark(20°C) conditions. After 10 days, the seedlings were then transplanted into pots containing a mixture of soil and vermiculite (1:1, w/w) under the same condition.

The highly aggressive defoliating isolate Vd991 of *V*. *dahliae* was cultured on potato dextrose agar (PDA) at 25°C for 7days, and then inoculated into Czapek liquid medium (0.3% NaNO_3_, 0.1% K_2_HPO_4_, 0.05% MgSO_4_·7H_2_O, 0.05% KCl, 0.001% FeSO_4_, 3% sucrose, pH 7.2). After culture for 7 days, the suspension was harvested by filtration through four layers of cheesecloth and then the culture density was adjusted to 10^7^ conidia/mL before further use.

Four-week-old seedlings of *C*. *komarovii* were gently removed from the soil and cleaned with water. The seedlings were placed into the solutions containing 1 mM salicylic acid (SA) and 20 mM H_2_O_2_, respectively. For pathogen-infection treatment, the roots of the seedlings were inoculated with *V*. *dahliae* conidial suspension for 10 min. For mechanical wounding, a hemostat was pressed across the stem. Control samples were treated with sterile water. The seedlings subjected to different treatments were harvested at appropriate times,—frozen in liquid nitrogen and used for RNA extraction. Three replicate experiments were carried out.

### Gene cloning and sequence analyses

Total RNA from *C*. *komarovii* seedilings was isolated using an extraction kit (Promega, WI, USA). Polyadenylated mRNA was obtained using a PolyATract mRNA Isolation System (Promega). A cDNA library was constructed as previously described [28] and propagated on 140-mm plates approximately 10^6^ clones were obtained. The conserved region of *AtSNAP33* isolated from Arabidopsis [1], was labeled with ^32^P-dUTP and used as probe for positive plaques by in situ hybridization. Four positive plaques were obtained after three screening rounds. The corresponding clones were sub-cloned into pBlueScript II SK (+) using the in vivo excision protocol provided by the manufacturer (Stratagene, USA).

The nucleotide sequence of *CkSNAP33* and the deduced amino acid sequence were investigated via an NCBI/Blast search. The theoretical isoelectric point (pI) and the molecular mass were calculated by ProtParam; the transmembrane domain of CkSNAP33 was predicted using the TMHMM online tool. A phylogenetic analysis was carried out in MEGA 5.1. Clustal Omega and SMART were used for multiple sequence alignment and domain prediction, respectively.

### *CkSNAP33* expression analysis

Total RNAs from *C*. *komarovii* seedlings in different treatments were isolated using RNA extraction Kit (Promega). RNA quality was evaluated using agarose gel electrophoresis and a NanoDrop 2000 spectrophotometer (ThermoFisher Scientific, Waltham, MA, USA). In total, 2 μg total RNA was reverse-transcribed to first-strand cDNA by High Capacity RNA-to-cDNA kit (Applied Biosystems, CA, USA). Real-time PCR was conducted to detect the *CkSNAP33* transcript level, using *CkEF-1-α* (HQ849463) from *C*. *komarovii* as the reference gene with the specific primers qEF1α-F/qEF1α-R [25]. A pair of primers (q33-F/q33-R) was designed to amplify a 178-bp fragment of *CkSNAP33* spanning the first intron (the sequences of the primers mentioned here are listed in S1 Table). For real-time PCR, a SYBR *Premix Ex Taq*^*™*^ *II kit* (TaKaRa, Dalian, China) and an ABI 7500 thermocycler (Applied Biosystems) were used for quantifying gene expression. PCR was performed in a 20-μL volume under the following conditions: 95°C denaturation for 30 s, followed by 40 cycles of 95°C for 5 s and 60°C for 34 s. The relative level of *CkSNAP33* expression was determined using the comparative 2^-ΔΔCt^ method. Three independent replicates of each PCR assay were performed.

### Generation of transgenic Arabidopsis plants

The full-length *CkSNAP33* cDNA fragment was amplified using primers ZW33-F/ZW33-R with the XbaI/SalI restriction sites at the 5′ and 3′ ends, respectively. The resulting PCR fragment was inserted into a modified pCAMBIA 1300 vector with a hygromycin phosphotransferase (*hpt II*) gene and a green fluorescent protein (GFP) gene under the control of a super promoter [29]. The recombinant construct vector (S5A Fig) was introduced into Arabidopsis Columbia ecotype via *Agrobacterium tumefaciens* (strain GV3101) transformation. Transgenic Arabidopsis seeds were screened on MS plates containing 25 μg/mL hygromycin B and then verified by PCR analysis of genomic DNA using the vector-specific primers 1300-F/1300-R. T3 generation seedlings were used for further experiments.

### Subcellular localization

The Super 1300-*CkSNAP33-GFP* plasmid was precipitated onto gold beads and transformed into onion epidermal cells by microprojectile bombardment using PDS-1000/He^™^ (Bio-Rad, CA, USA). Subcellular localization of fluorescent protein was observed at 488 nm by confocal laser scanning biological microscope FLUOVIEW FV1000 (OLYMPUS, Tokyo, Japan). Plasmolysis was induced by incubating samples in 0.8 M mannitol for 10 min.

### Morphological examination of transgenic plants

Arabidopsis seedlings were grown in a vertical orientation on MS plates for 15 days and root lengths were measured using vernier calipers (Qualitot 141–494, Guilin, China). Four-week-old Arabidopsis plants were grown in pots and were photographed with a digital camera (Nikon D90, Tokyo, Japan). The fresh weight of the plant aerial portions was measured using an analytical balance (Ohaus AR2130, NJ, USA), and the leaf area of rosette leaves was computed using coordinate paper (Baolishi, Zhejiang, China) and Photoshop CS5 (Adobe Systems, San Jose, CA, USA).

### *V*. *dahliae* inoculation and disease investigation

For *V*. *dahliae* inoculation of Arabidopsis, three-week-old seedlings were removed from the soil, washed with water, and dried briefly with blotting paper to remove excess water. The roots were then immersed in a beaker containing a fresh spore suspension (10^7^ conidia/mL) for 10 min. The seedlings were transplanted into fresh soil immediately and kept in a high-humidity environment for 12 h at 25°C. Seedlings in the control group were mock-treated with sterile water. The disease index (DI) was measured periodically for 21days post-inoculation (dpi).

The quantification of *V*. *dahliae* biomass was performed as previously described [30]. The plants at 21 dpi were removed from the soil and ground to powder in liquid nitrogen; genomic DNA was extracted by the cetyltrimethyl ammonium bromide (CTAB) method and was quantified by NanoDrop 2000 spectrophotometer (ThermoFisher Scientific) and agarose gel electrophoresis. The biomass at the DNA level was determined by real-time PCR as described above. The primers used for the quantification of *V*. *dahliae* were qVd-F/qVd-R. *AtEF1-α* (NM_100666.3) expression level was used as the internal standard to normalize differences in DNA template amounts (the primer sequences are given in S1 Table).

Real-time PCR was performed as described above to determine the transcription level of the genes pathogenesis-related protein 1 (*PR1*) and pathogenesis-related protein 5 (*PR5*) in Arabidopsis infected with *V*. *dahliae* at 6 dpi. The specific primers for *PR1* and *PR5* were qPR1-F/qPR1-R and qPR5-F/qPR5-R, respectively. *AtEF1-α* was used as the endogenous control and was detected using the primer pair AtEF1α-F/AtEF1α-R (the sequences of the primers mentioned here are listed in S1 Table).

### Histological detection of cell death and callose deposition in leaves

To investigate the resistance mechanism induced by expression of *CkSNAP33* in transgenic Arabidopsis, leaves were inoculated with 5-μL of *V*. *dahliae* suspension (10^7^ conidia/mL) and incubated at 25°C in a moist chamber. The infected leaves were then stained with aniline blue for callose deposition at 24 hours post-inoculation (hpi) [31] and subjected to trypan blue staining to show fungal structure and cell death at 48 hpi [32, 33]. Callose deposition was monitored by fluorescence microscopy (Nikon C1) and cell death was observed and imaged under an optical microscope (Nikon ECLIPSE Ti, Tokyo, Japan).

### Statistical analysis

All experiments and measurements were performed with three replicates per treatment. Statistical analyses were performed with SPSS.16 using one-way ANOVA. Data are presented as mean ± SE. Significant differences were determined at the 5% level of significance and asterisks are used to indicate p-values: *p < 0.05, **p < 0.01.

## Results

### Characterization of CkSNAP33

**Fig 1:**
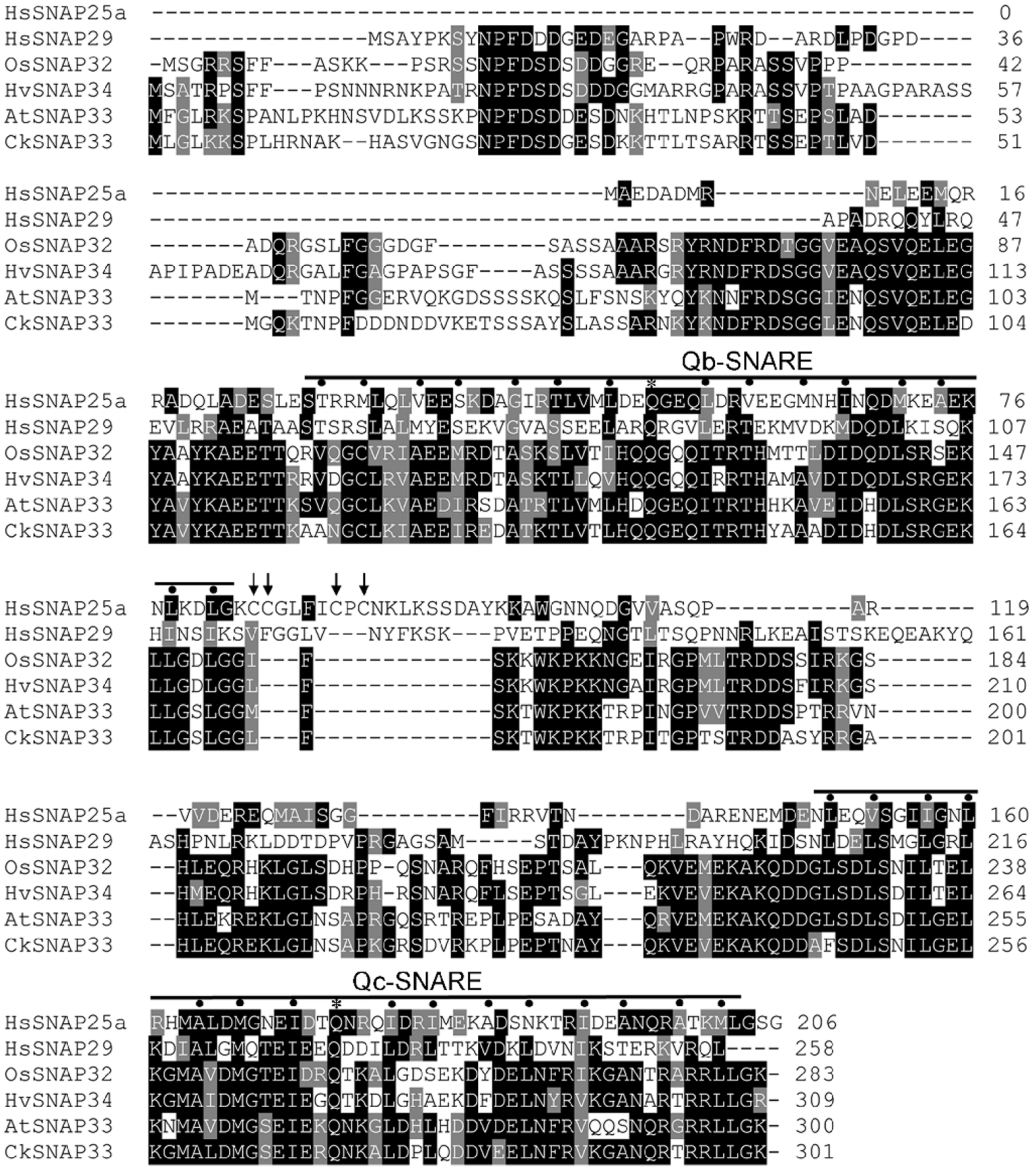
Protein sequence alignment of CkSNAP33 with other SNAP25 proteins. HsSNAP25a (AAH10647.1) and HsSNAP29 (O95721.1) from *Homo sapiens*, OsSNAP32 (AAW82752.1) from *Oryza sativa* L., HvSNAP34 (AAP79417.1) from *Hordeum vulgare*, and AtSNAP33 (Q9S7P9.1) from *Arabidopsis thaliana*. Conserved residues are shaded in black and similar residues in gray. Positions in Qb-and Qc-SNARE domains that contribute to stabilizing ionic or hydrophobic interaction with other SNARE proteins are marked using asterisks and dots, respectively. The four cysteine residues involved in palmitoylation and membrane association of SNAP25 are indicated using arrow. Multiple amino acid sequence analyses were performed using Clustal Omega and the multiple alignment file was shaded using the BoxShade program.

**Fig 2:**
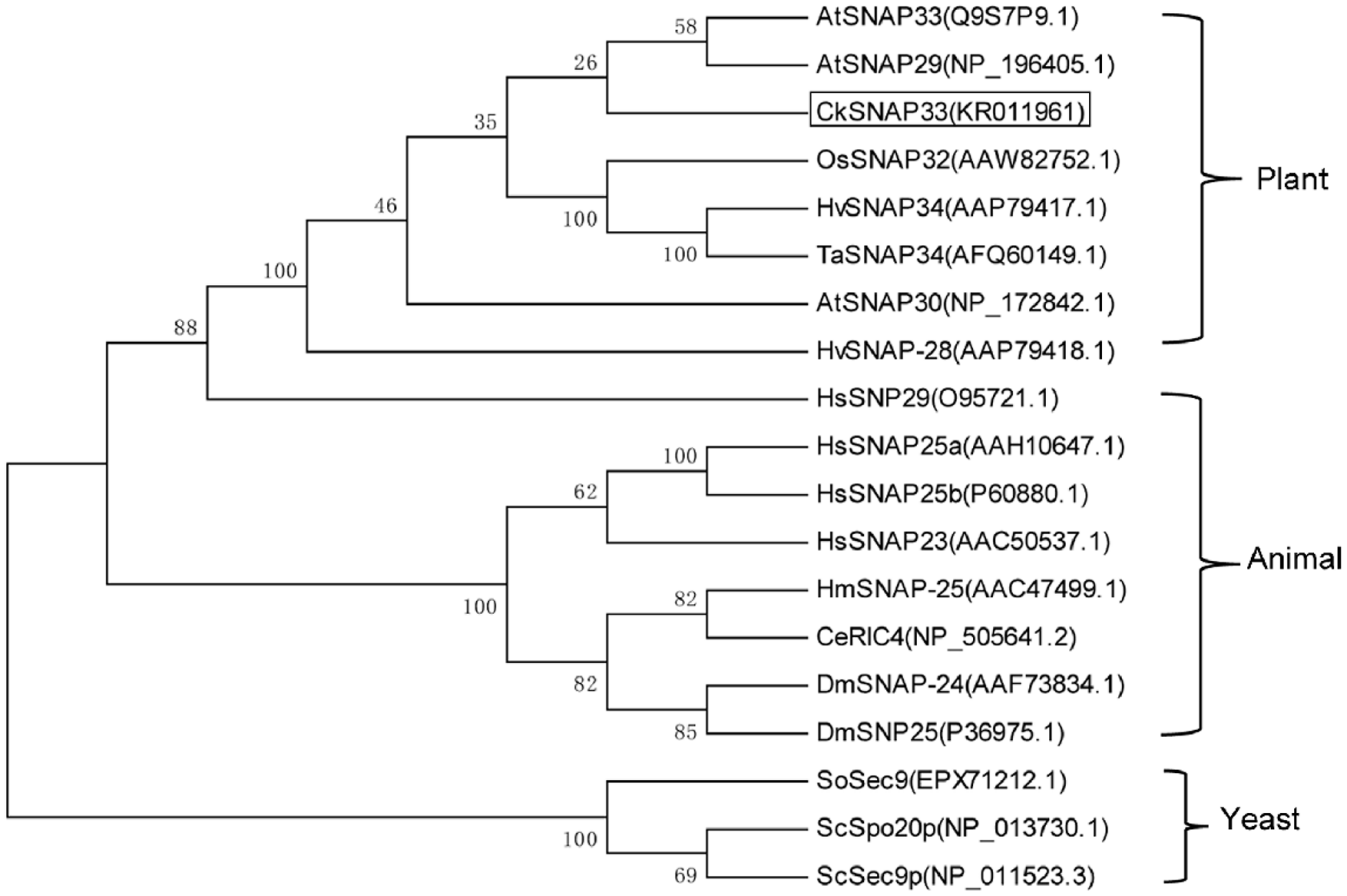
Phylogenetic tree of SNAP25 proteins. The phylogenetic tree was constructed using the neighbor-joining method with MEGA 5.1, and bootstrap values from 1,000 replicates are indicated at the nodes.

The target cDNA was obtained from *C*. *komarovii* using colony in situ hybridization, and designated *CkSNAP33* (GenBank accession number: KR011961). The full-length cDNA of *CkSNAP33* is 1298 bp long, including a 906 bp ORF that encodes a putative protein of 301 amino acids (S1 Fig) with a predicted pI of 6.63 and a calculated molecular weight of approximately 33.28 kD. It has a genomic DNA sequence of 1666 bp, with five exons and four introns (S2 Fig); this is similar to exon—intron structure of OsSNAP32 [20]. A TMHMM analysis indicated that CkSNAP33 did not have a transmembrane domain (S3 Fig). Multi-sequence alignment analysis of CkSNAP33 with other SNAP25-type proteins revealed that it shares high identity with AtSNAP33 (68.75%), AtSNAP30 (58.54%), OsSNAP32 (55.48%), and HvSNAP34 (53.38%). CkSNAP33 also contains a Qb-SNARE domain from Ala-102 to Gly-169 and a Qc-SNARE domain from Ala-231 to Leu-298 (Fig 1); these domains are the characteristic dual heptad repeat SNARE motifs of SNAP25 proteins [34]. Some SNAP25-type proteins contain palmitoylation modifications at a conserved cysteine cluster to mediate membrane anchorage; these four cysteine residues do not exist in CkSNAP33, other plant SNAP25-type proteins, or HsSNAP29 (Fig 1). The phylogenetic tree of the SNAP25 homologies from various organisms showed three differentiated main branches (plants, animals, and yeasts), and CkSNAP33 was clustered into a clade that included AtSNAP33 (NP_200929.1) and AtSNAP29 (NP_196405.1) (Fig 2). Tissue-specific expression of *CkSNAP33* in *C*. *komarovii* showed higher in roots than that in stems and leaves (S4 Fig).

### CkSNAP33 expression is induced by H_2_O_2_, SA, *V*. *dahliae*, and wounding

**Fig 3:**
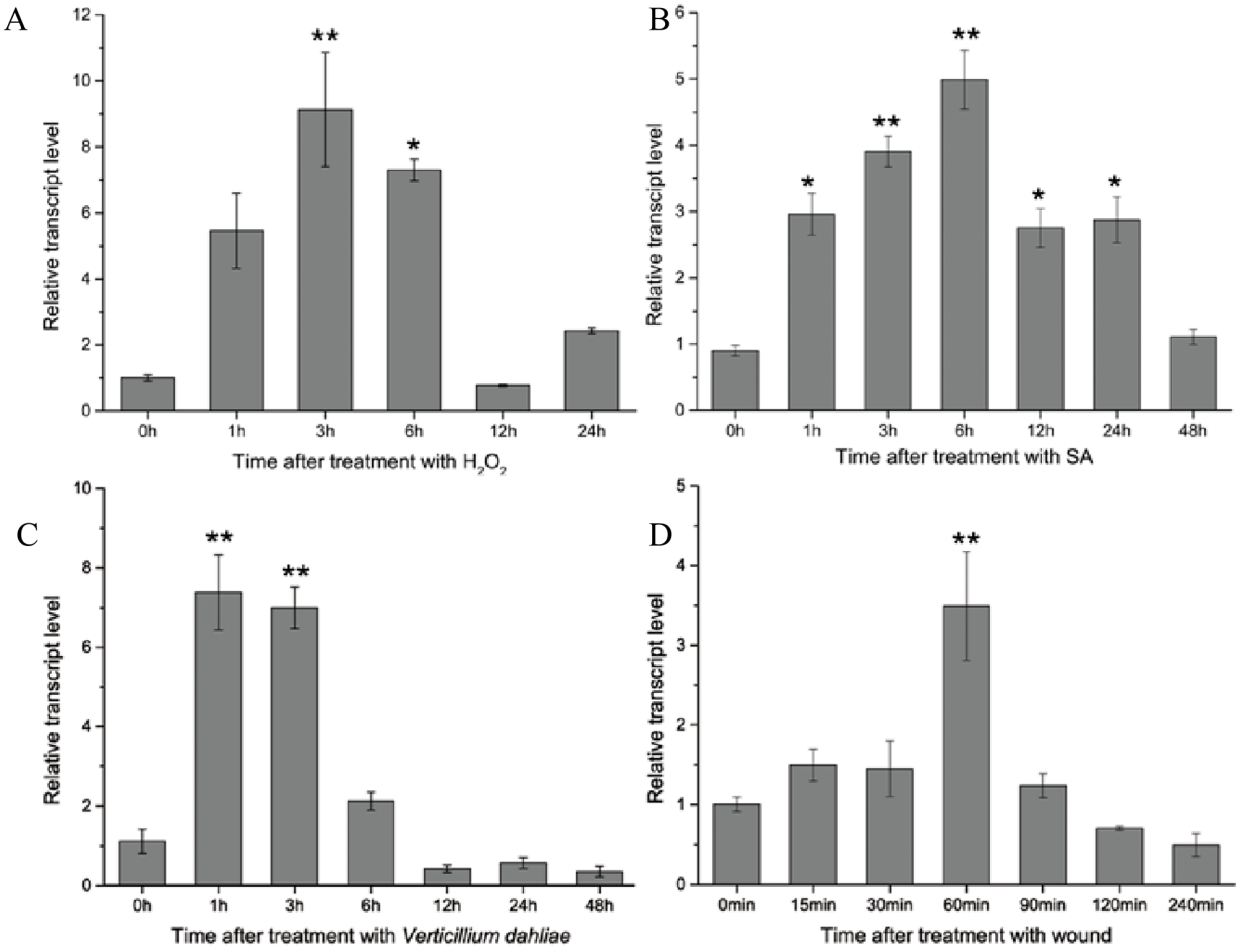
The expression patterns of *CkSNAP33* under different conditions. (A) 20 mM H_2_O_2_. (B) 1 mM salicylic acid (SA). (C) *Verticillium dahliae*. (D) Wounding. Data were collected from three independent biological repeats. Results are expressed as mean ± standard error (SE; n = 3). Asterisks show significance difference (* p < 0.05; **p < 0.01).

*CkSNAP33* transcript levels in *C*. *komarovii* seedlings were measured under various stresses. During H_2_O_2_ treatment, *CkSNAP33* was gradually elevated and reached its peak at 3 h, and decreased thereafter (Fig 3A). *CkSNAP33* transcription was immediately up-regulated after SA treatment and maintained at a high level until 48 h (Fig 3B). In seedlings inoculated with *V*. *dahliae*, *CkSNAP33* transcription was significantly up-regulated at 1 and 3 hpi and gradually declined at 6 and 12 hpi (Fig 3C). *CkSNAP33* transcript level reached a maximum at 60 min after wounding treatment (Fig 3D).

### CkSNAP33 is located at plasma membrane

**Fig 4:**
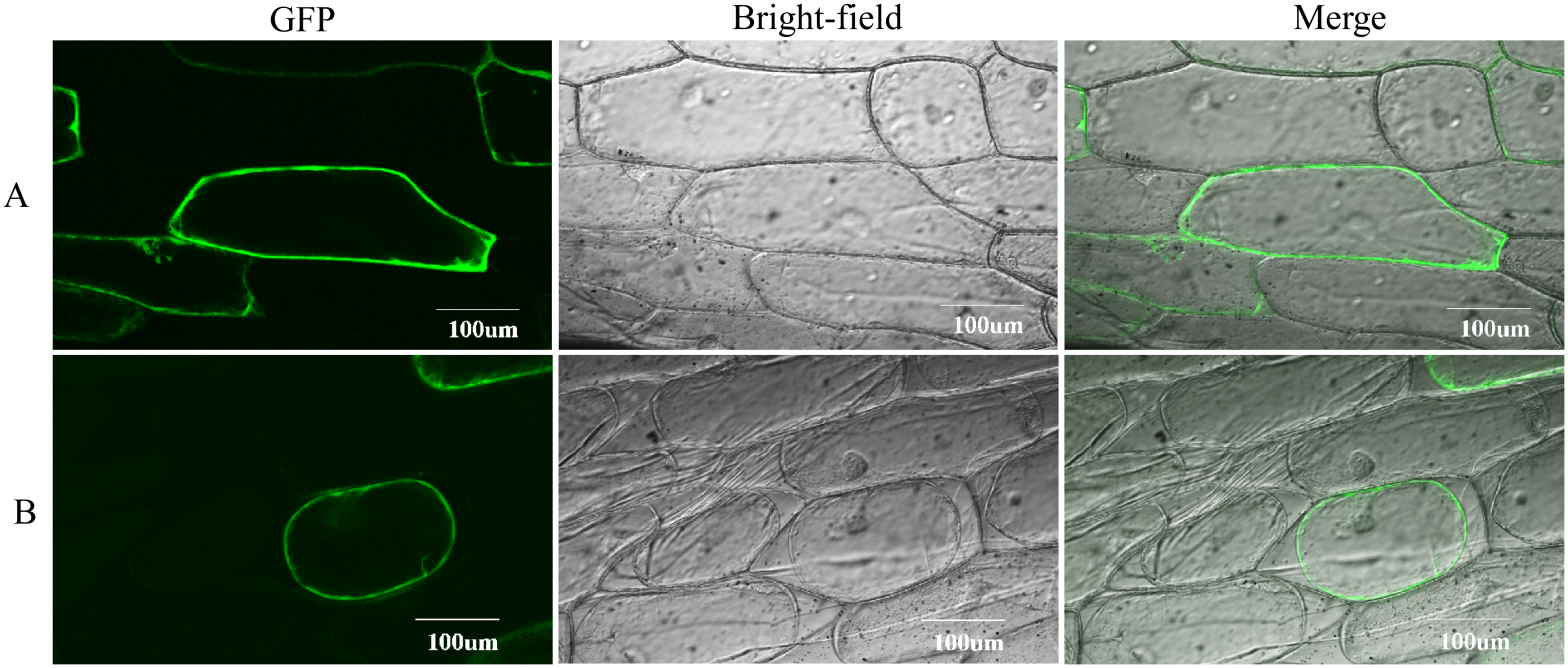
Subcellular localization of CkSNAP33-GFP fusion protein. (A) Confocal images of transformed onion epidermal cells. (B) Confocal images of transformed onion epidermal cells after plasmolysis.

The subcellular localization of CkSNAP33 was examined using CkSNAP33-GFP fusion protein expressed in onion epidermal cell (Fig 4). The GFP fluorescence was observed at the cell wall or at the plasma membrane (Fig 4A). The samples were treated with 0.8 M mannitol to differentiate between the plasma membrane and the cell wall localization; the GFP fluorescence after plasmolysis revealed that CkSNAP33 was localized at plasma membrane (Fig 4B).

### Overexpression of CkSNAP33 promotes the growth of Arabidopsis plants

A total of 11 independent Arabidopsis lines overexpressing *CkSNAP33* were obtained by selection for hygromycin B resistance and genomic DNA-PCR analysis (S5B Fig). Real-time PCR suggested that a range of expression levels of *CkSNAP33* was present in the transgenic lines (S5C Fig). L12 showed the highest CkSNAP33 expression, while L3 and L10 showed an intermediate level of expression. L12 and L3 were selected for further functional analysis.

**Fig 5:**
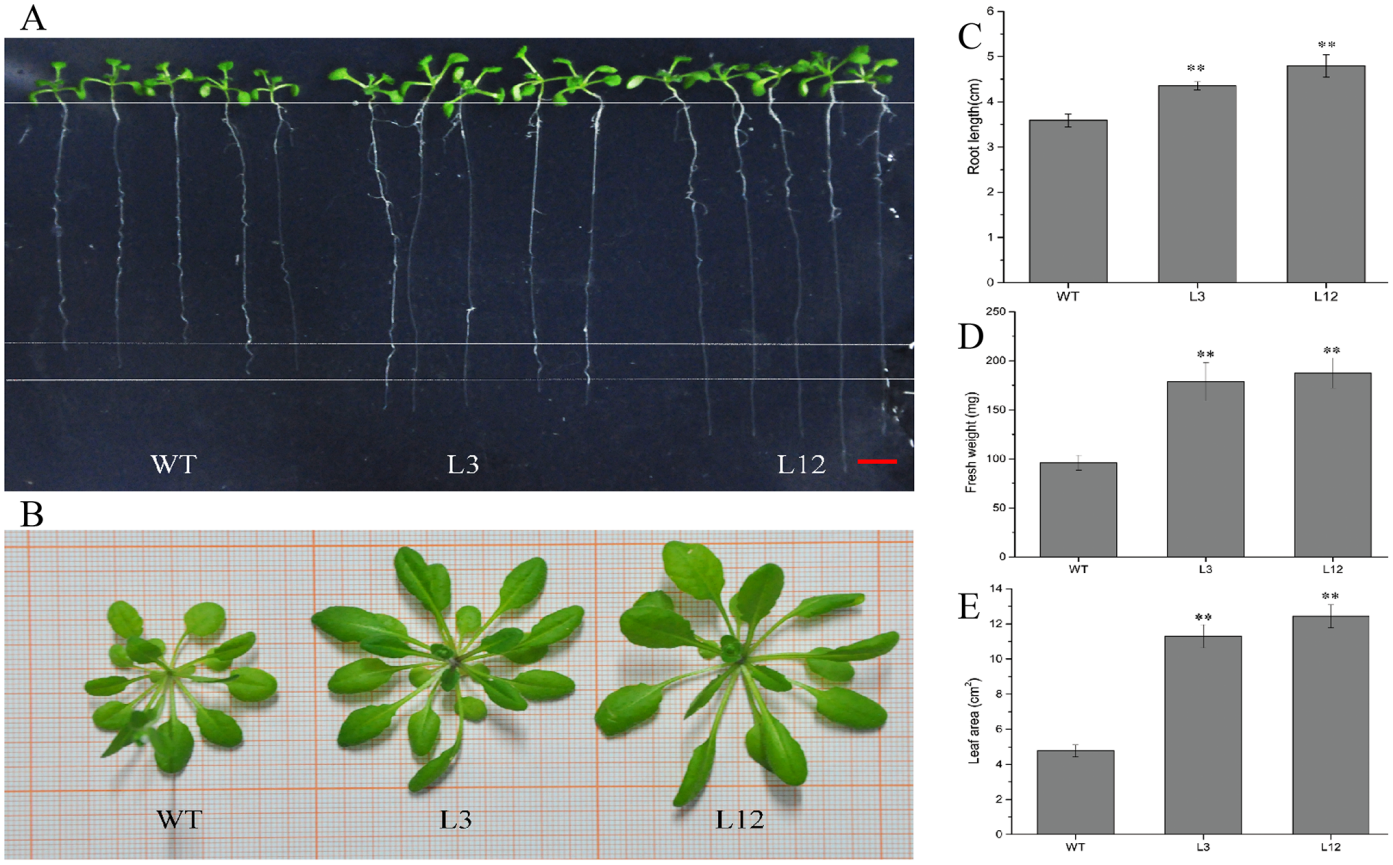
Developmental phenotypes of transgenic and wild type (WT) plants. (A) 15-day-old Arabidopsis seedlings of WT and representative *CkSNAP33* overexpression lines (L3, L12) grown on vertically orientated Murashige-Skoog plates. Scale bar represents 5 mm. (B) Morphology of the rosettes of 4-week-old Arabidopsis plants. (C) Root length of 15-day-old seedlings. (D) Fres weight of 4week-old seedlings. (E) Leaf area of 4-week-old seedlings. Data are mean ± SE (n = 3). Asterisks show significant differences (**p < 0.01).

The L3 and L12 lines had longer roots than WT seedlings at 15 days (Fig 5A and 5C). In four-week-old seedlings grown in soil, transgenic plants were generally larger than WT (Fig 5B). The fresh weight of transgenic plants was about 1.9-fold greater than that of WT (Fig 5D) and leaf area in transgenic plants was about 2.36–3.10-fold greater than that of WT (Fig 5E).

### Overexpression of CkSNAP33 confers Arabidopsis enhanced disease resistance to *V*. *dahliae*

**Fig 6:**
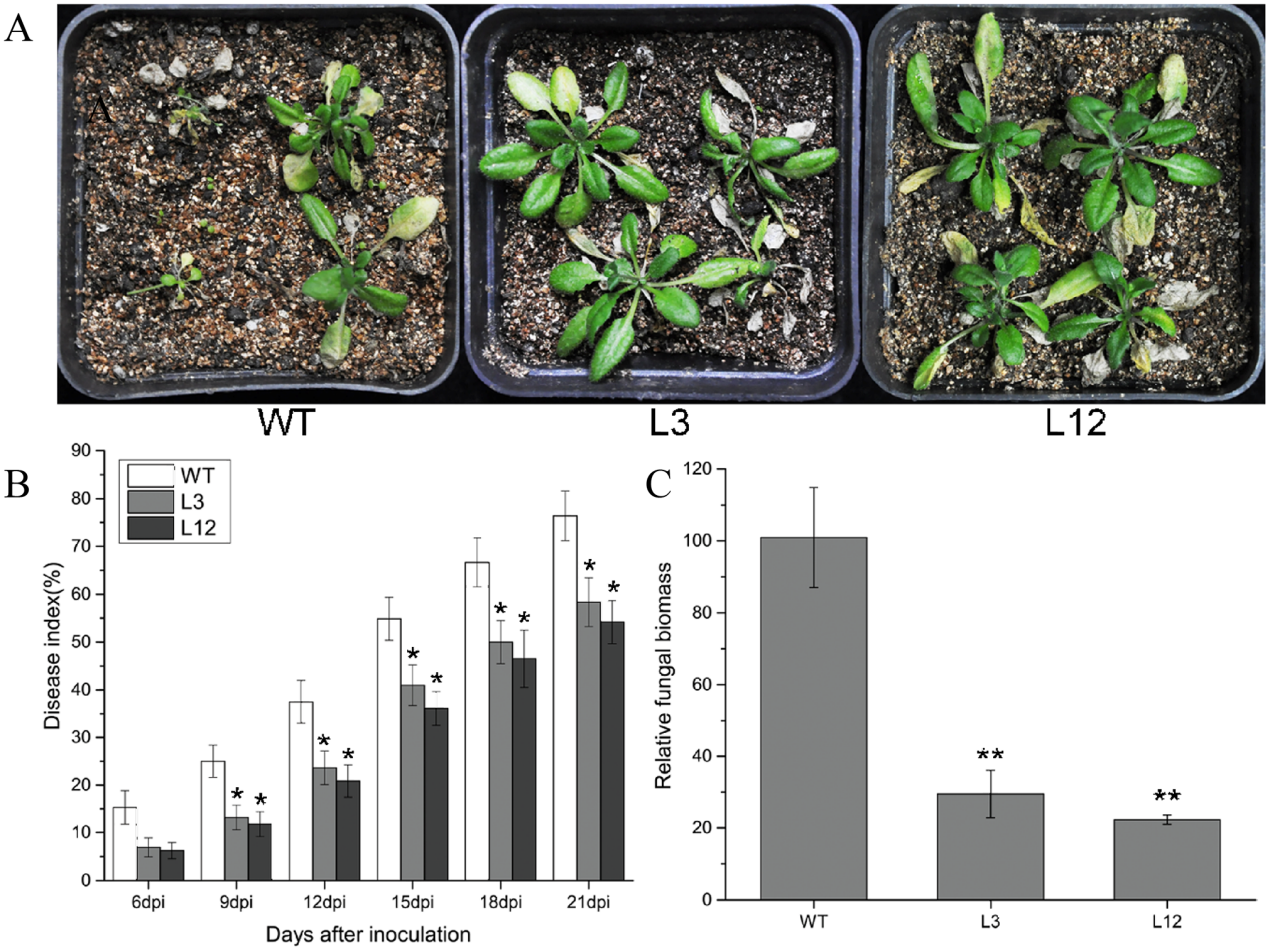
Transgenic expression of *CkSNAP33* enhances resistance to *Verticillium dahliae*. (A) Disease phenotypes in WT and two transgenic plants (L3, L12) infected by *V*. *dahliae*. Images were captured at 21 dpi. (B) Disease index of WT and transgenic Arabidopsis plants at the indicated days after inoculation. Data are means ± SE (n = 3). (C) Fungal biomass in infected Arabidopsis plants at 21 dpi. Results are mean ± SE (n = 3). Asterisks represent significance differences (*p < 0.05; **p < 0.01).

*CkSNAP33* transgenic lines and WT Arabidopsis were inoculated with *V*. *dahliae*. At 21 dpi, WT plants showed serious wilting; whereas plants of the transgenic lines showed less intense symptoms (Fig 6A). Disease indexes (DI) were recorded from 6 dpi to 21 dpi (Fig 6B). At 21 dpi, WT plants showed a higher DI (76.39) compared with plants of the transgenic L3 and L12 lines (58.33 and 54.17, respectively) (Fig 6B). The relative fungal biomass assay gave consistent results to the DI analysis and *V*. *dahliae* biomass in the transgenic lines was lower than that in WT (Fig 6C), implying that *CkSNAP33* overexpression enhanced Verticillium wilt resistance in *Arabidopsis*.

**Fig 7:**
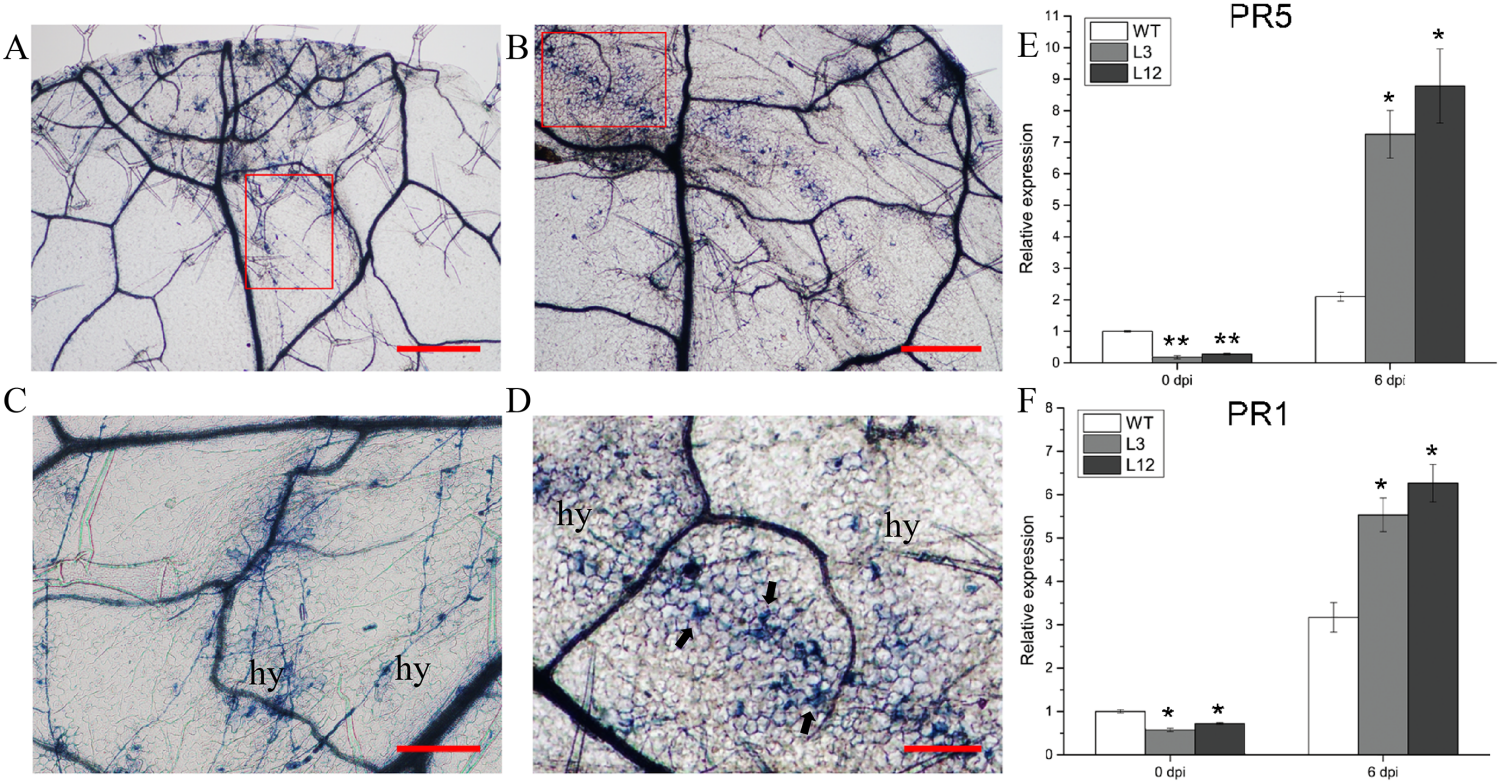
Inducing the hypersensitive response (HR) and pathogenesis-related genes in *CkSNAP33* over-expressing Arabidopsis. Trypan blue staining of leaf sections from WT (A) and transgenic plants (B) at 6 dpi with *V*. *dahliae*. Scale bar represents 500 μm. (C) Normal growth of *V*.*dahliae* hyphae (hy) across the leaf surface of WT. Close-up of the disease lesion regions from (A). Scale bar represents 200 μm. (D) *V*.*dahliae* induced programmed cell death (arrows) around the hyphae (hy). Close-up of the disease lesion regions from (B). Scale bar represents 200 μm. Real-time PCR analysis for the transcript levels of *PR5* (E) and *PR1* (F) in WT and transgenic lines before (0 dpi) and 6 days after inoculation with *V*. *dahliae* (6 dpi). Data are mean ± SE (n = 3), asterisks represent significant differences (*p < 0.05; **p < 0.01).

Trypan blue staining of leaf tissues at 6 dpi was used to identify fungal hyphae in leaves of WT and transgenic plants (Fig 7A and 7B). Leaves from WT showed more free hyphae (Fig 7A and 7C). Leaves from plants of the L3 and L12 lines showing *CkSNAP33* overexpression showed trailing cell death around the hyphae (Fig 7B and 7D). Cell death in the *CkSNAP33* transgenic lines appeared to be associated with the enhanced resistance. Real-time PCR results for expression of PR genes indicated that *PR1* and *PR5* transcript levels increased in infected Arabidopsis plants and these levels in L3 and L12 were considerably higher than those in WT at 6 dpi, especially for *PR5* (Fig 7E and 7F).

**Fig 8:**
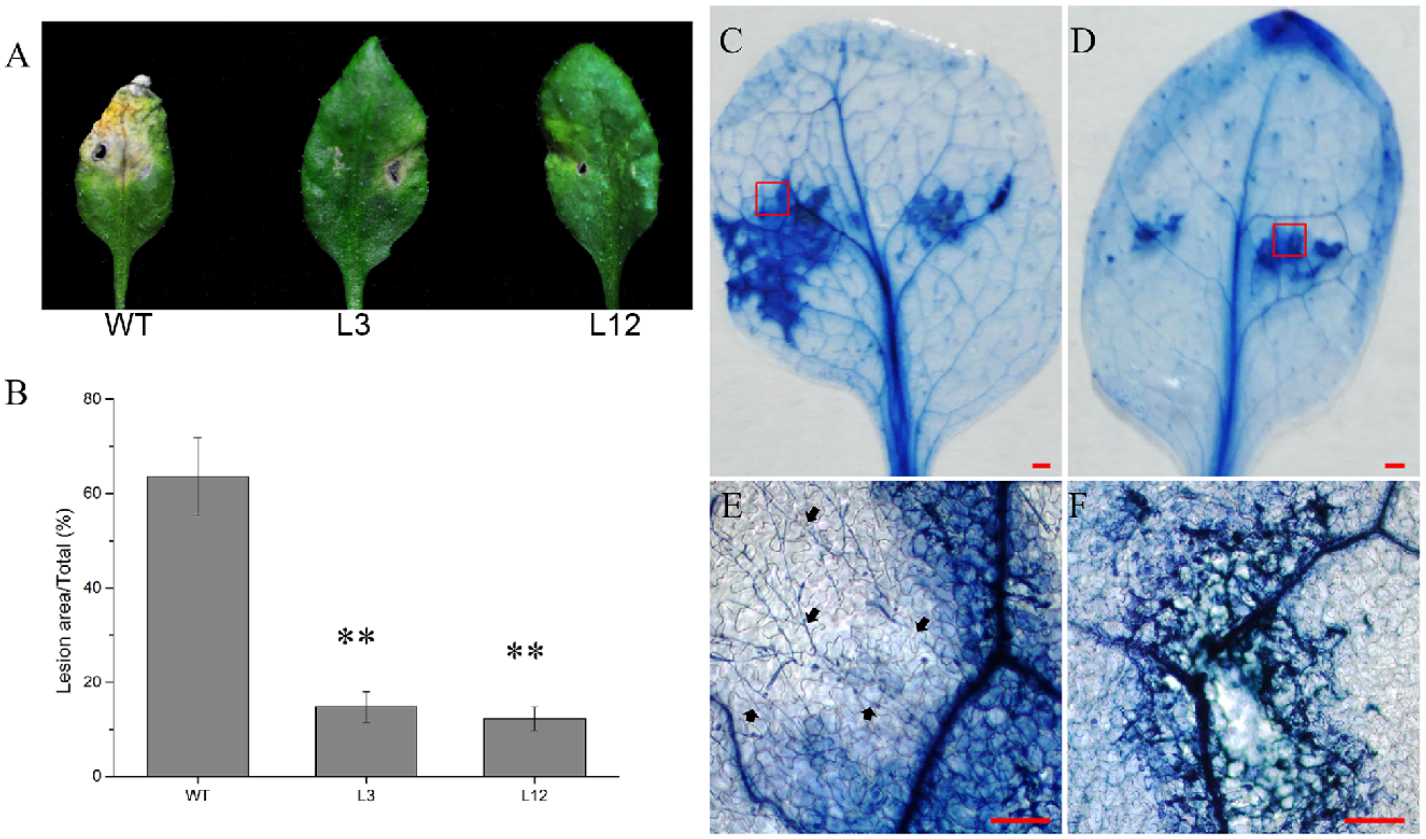
Over-expression of *CkSNAP33* in Arabidopsis hinders the expansion of *V*.*dahliae* in leaves. Disease symptoms (A) and lesion size (B) in the WT and the transgenic lines at 6 dpi, Data are means ± SE (n = 3) and asterisks represent significant differences (**p < 0.01) in (B). Trypan blue staining of *V*. *dahliae*-infected leaves of WT (C) and transgenic lines (D); scale bars represent 3 mm. (E) and (F) Close-ups of the disease areas from (C) and (D). The growth of free hyphae (arrows) exceeds the cell death zone in (E). Scale bars represent 50 μm.

To further investigate the function of CkSNAP33 in resistance to *V*. *dahliae*, 5 μL conidial suspension was dropped onto Arabidopsis leaves. At 6 dpi, WT leaves showed extensive necrosis; by comparision, slight etiolation of the leaves was observed at the inoculation site in transgenic lines (Fig 8A and 8B). The transgenic plants showed clear resistance to *V*. *dahliae* at the inoculation site, especially plants in L12.

Trypan blue staining at 48 hours post inoculation (hpi) revealed that WT plants not only showed large necrotic patches but the hyphae also extended into the adjacent areas (Fig 8C and 8E). In contrast, the transgenic lines showed lower necrosis and no hyphae were detected (Fig 8D and 8F), indicating that hypersensitive reaction (HR) induced cell death is exhibited in transgenic lines, which is capable of restricting the development of pathogens [35].

**Fig 9:**
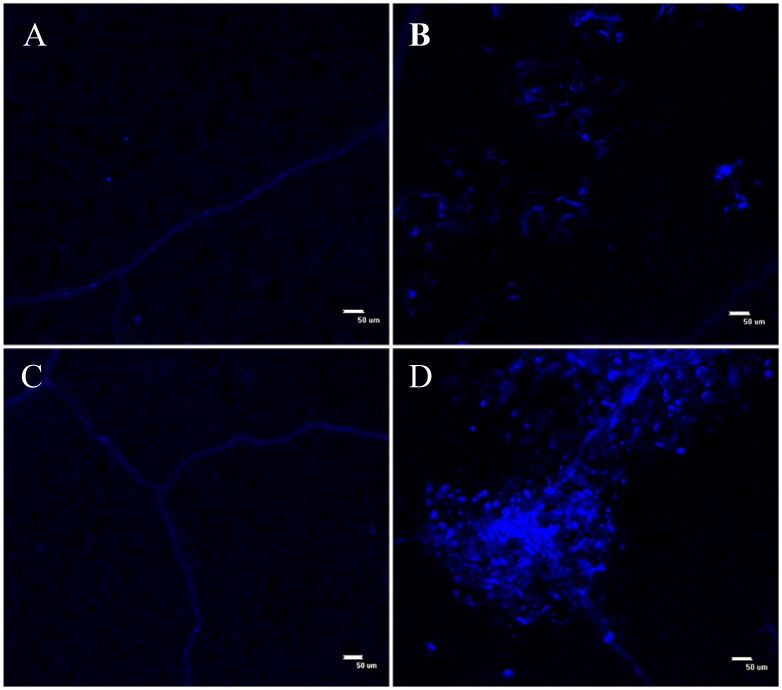
Callose deposition in *Verticillium dahliae*-infected leaves of WT and CkSNAP33 over-expressing plants. (A) Control WT leaf. (B) *V*. *dahliae* WT infected leaf. (C) Control transgenic leaf. (D) *V*. *dahliae* infected transgenic leaf. Scale bar represents 50 μm.

SNARE proteins are involved in the resistance to penetration of pathogens at the cell wall [22]. The results of the aniline blue assay at 24 hpi indicate that the transgenic lines accumulated more callose than WT leaves at infection sites (Fig 9). Overexpression of *CkSNAP33* might increase the transport of callose synthases to the infection sites and enhance callose accumulation, then inhibit invasion by *V*. *dahliae*.

## Discussion

In eukaryotic cells, SNARE proteins can form a complex that allows membrane fusion between vesicles, organelles, and plasma membrane. All SNAREs contain a sequence termed the SNARE motif that is predisposed to form coiled-coils [2]. The neuronal SNARE core complex contains motifs of syntaxin 1A (Qa-SNARE), SNAP25 (Qb- and Qc- SNARE) and synaptobrevin/VAMPII(R-SNARE) and forms a parallel four-helix bundles[2, 36]. The present study cloned and characterized a SNAP25 homolog from *C*. *komarovii*, termed *CkSNAP33*.Similarly to other SNAP25 proteins, CkSNAP33 contains an N-terminal Qb-SNARE motif with Gln-141 and a Qc-SNARE motif with Gln-270 in the C-terminal region, which contribute to the formation of the “zero ionic layer” with Qa-SNARE and R-SNARE. We showed that CkSNAP33 is located at the plasma membrane; this intracellular distribution is consistent with the localizations of several SNAP25-type proteins in plants [1, 20]. However, unlike syntaxin and synaptobrevin, SNAP25-type proteins lack transmembrane domain at C-terminal [37]. However, some SNAP25 homologs are associated with membranes and these associations appear to depend on post-translational palmitoylation of cysteine residues located at the center of the peptide sequence [38, 39]. However, the cysteine cluster is not present in CkSNAP33, AtSNAP33, HsSNAP29 nor HsSNAP34. Whether the cysteine at position 120 of SNAP33 is modified by lipid at its only or hydrophobic post-translational modifications at another residue remains to be determined.

Expression of SNAP25-type genes in plants can be induced by a variety of defense-related chemicals and pathogens. For instance, exogenous SA and thiadiazole-7-carbonic acid S-methyl ester (BTH) induce expression of *AtSNAP33* [21]. *OsSNAP32* expression is significantly activated by H_2_O_2_ in rice [20]. In *C*. *komarovii*, we showed here that transcript levels of *CkSNAP33* also increased during treatment with H_2_O_2_ and SA. This finding indicated that *CkSNAP33* expression was inducible by a signal molecule such as SA and H_2_O_2_ and that *CkSNAP33* was involved in the defense-signaling network in the plant. Furthermore, we found that the transcription of *CkSNAP33* was induced by *V*. *dahliae*. *OsSNAP32* can also be activated by rice blast (*Magnaporthe grisea*) [40] and the expression of *AtSNAP33* is induced by several pathogens, including *Plectosporium tabacinum*, *Peronospora parasitica*, and *Pseudomonas syringae* pv *tomato* [21]. *AtSNAP33* expression is regulated by SA-dependent and SA-independent pathways after pathogen infection, [21], our results suggested that *CkSNAP33* was involved in an Arabidopsis defense response against *V*. *dahliae* through either the SA or H_2_O_2_ signaling pathway.

SNAP33 is essential for the growth and development of plants [1, 19]. *AtSNAP33* shows high expression in various tissues, including roots, stems, leaves, and flowers [1, 4]. In rice, *OsSNAP32* is highly expressed in leaves and expression in roots is low [20]. The high level of expression of *CkSNAP33* in roots of *C*. *komarovii* may be associated with the particular root traits and small leaves of desert plants, and this observation suggested that *CkSNAP33* is preferentially expressed in vigorous tissues in plant. We found that the transgenic Arabidopsis lines that overexpressed *CkSNAP33* had longer roots and were physically larger than WT plants. AtSNAP33 interacts with the cytokinesis-specific syntaxin KNOLLE (KN) at cell plate formation in plant cytokinesis [1]. This indicates that *CkSNAP33* overexpression might increase membrane fusion associated with cytokinesis in transgenic Arabidopsis. However, the mechanisms through which overexpression of *CkSNAP33* can promote the growth of Arabidopsis plant remain to be determined.

Penetration resistance and programmed cell death are two major strategies in plants to restrict the invasion of biotrophic fungal pathogens [41].Here, we found a reduced disease phenotype and disease index, low pathogen biomass, and increased levels of *PR1* and *PR5* transcripts in transgenic Arabidopsis carrying *CkSNAP33* indicating enhanced resistance to *V*. *dahliae*. Papillae, callose-containing cell-wall appositions, are effective barriers during the relatively early stages of pathogen invasion [42, 43] and the papillae in grapevine can block penetration and haustoria formation against powdery mildew[41]. Barley HvSNAP34 protein and its binding partner ROR2 mediate penetration resistance against *Blumeria graminis*.f.sp. *hordei* at the cell wall [22]. Furthermore, PEN1 and SNAP33 are incorporated into powdery mildew at the papillary matrix [31]. In our study, increased callose deposition was induced in the infected leaves of transgenic Arabidopsis compared to WT leaves, indicating that CkSNAP33 participated in papilla formation in Arabidopsis and was involved the early defense responses against *V*. *dahliae*. Plant pathogens are known to activate the H_2_O_2_ and SA pathways and trigger defense responses in the form of the hypersensitive response (HR), leading to hypersensitive cell death and systemic acquired resistance in the host plant [24, 44, 45]. The trypan blue assay showed HR-induced cell death occurred in transgenic lines at the infection site, and that this limited pathogen in the death cell zone. These results suggested that CkSNAP33 was involved in the early defense responses at the cell wall and that a defense signal activated the HR to enhance disease resistance in *Arabidopsis*.

In summary, CkSNAP33 is a ubiquitous synaptosomal-associated protein, belonging to SNAP25-type t-SNARE family. *CkSNAP33* overexpression promoted plant growth in transgenic Arabidopsis and enhanced disease resistance to *V*. *dahliae* through callose deposition and HR-induced cell death. This study contributes to the elucidation of the function of SNAP25 homolog proteins in pathogen defense. However, further research is necessary to define the role of CkSNAP33 in transport of defense signal molecules or products via membrane fusion.

## Supporting information

S1 Fig Nucleotide sequence and deduced amino acid sequence of *CkSNAP33*. The Qb-SNARE and Qc-SNARE domains are shown in gray. The amino acids highlighted in yellow are conserved glutamine residues of the Q-SNARE domains.
(TIF)

S2 Fig Schematic structure of CkSNAP33. (TIF)

S3 Fig CkSNAP33 lacks a transmembrane domain according to TMHMM analysis. (TIF)

S4 Fig Tissues specific expression pattern of *CkSNAP33* in *C*. *komarovii* by real-time PCR. Data were collected from three independent biological repeats. Results are shown as means ±SE (n = 3). Asterisks indicate significant differences (* p<0.05, **p<0.01).
(TIF)

S5 Fig Genetic transformation of Arabidopsis with *CkSNAP33* and identification of transgenic lines. (A) Schematic representation of the modified pCAMBIA 1300 vector with *CkSNAP33* under the control of a super promoter. (B) Genomic DNA-PCR analysis of genomic DNA from hygromycin B resistant lines. (C) Real-time PCR confirmed the expression of *CkSNAP33* in transgenic lines.
(TIF)

S1 Table List of primers used in this study. (DOCX)
