# Supplementary figures and images for: Identification of CkSNAP33, a gene encoding synaptosomal-associated protein from Cynanchum komarovii, that enhances Arabidopsis resistance to Verticillium dahliae
Source: PLoS One. 2017 Jun 2;12(6):e0178101. doi: 10.1371/journal.pone.0178101 (PMC5456056; doi:10.1371/journal.pone.0178101)

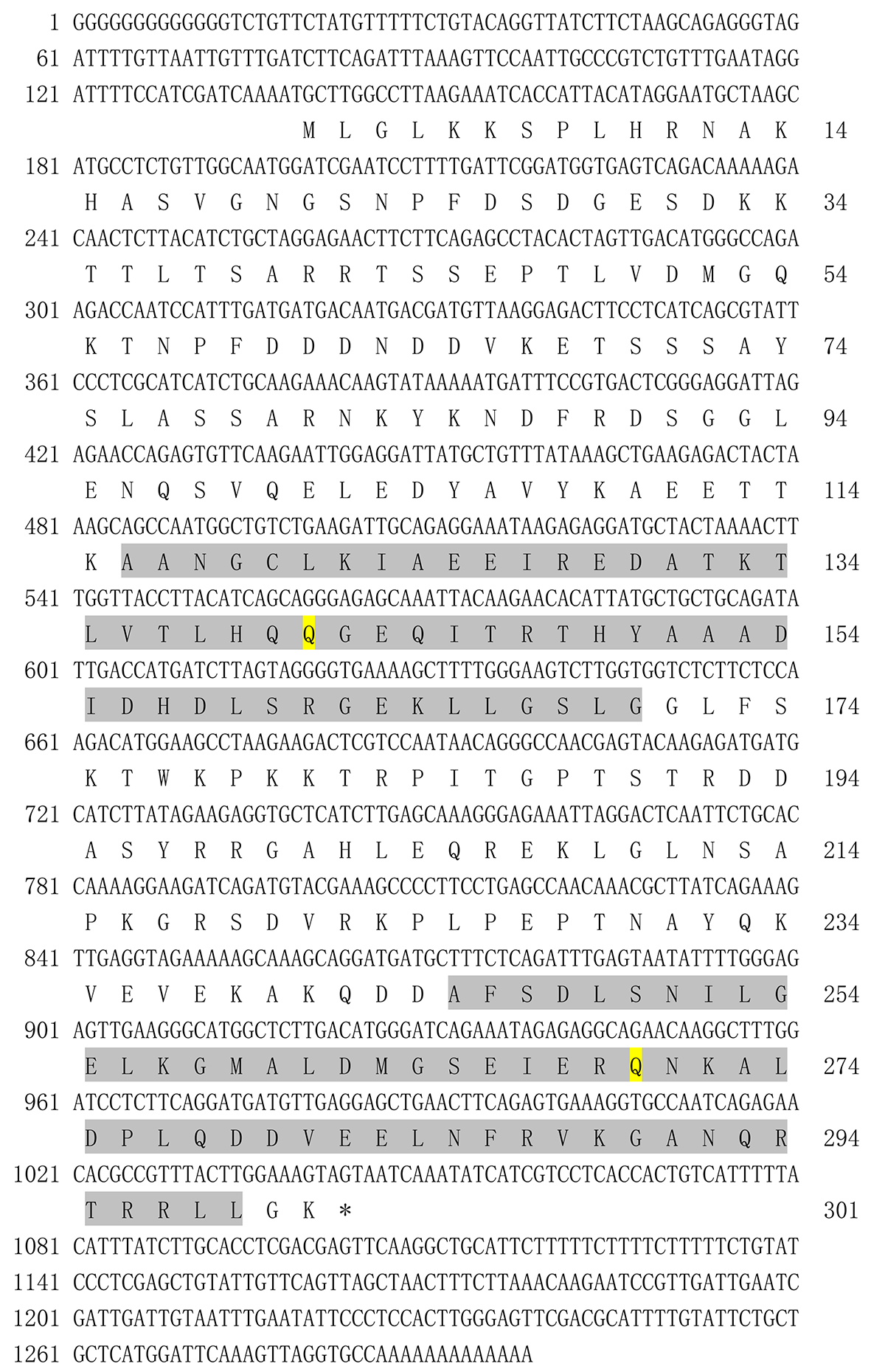

Supplement: S1 Fig — The Qb-SNARE and Qc-SNARE domains are shown in gray. The amino acids highlighted in yellow are conserved glutamine residues of the Q-SNARE domains. (TIF) [file pone.0178101.s001.tif]

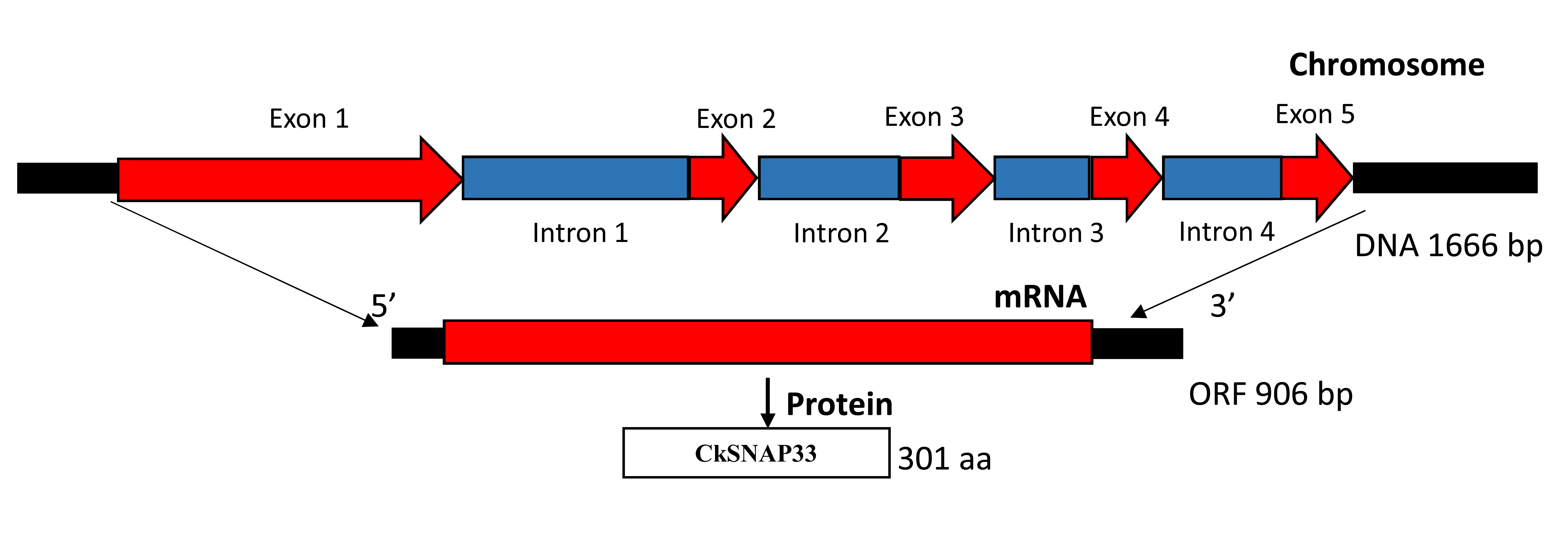

Supplement: S2 Fig — (TIF) [file pone.0178101.s002.tif]

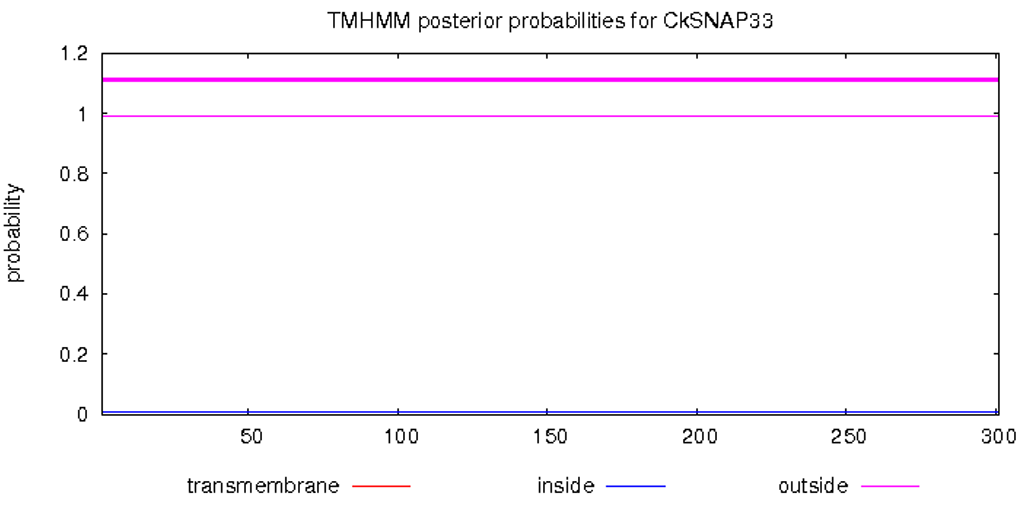

Supplement: S3 Fig — (TIF) [file pone.0178101.s003.tif]

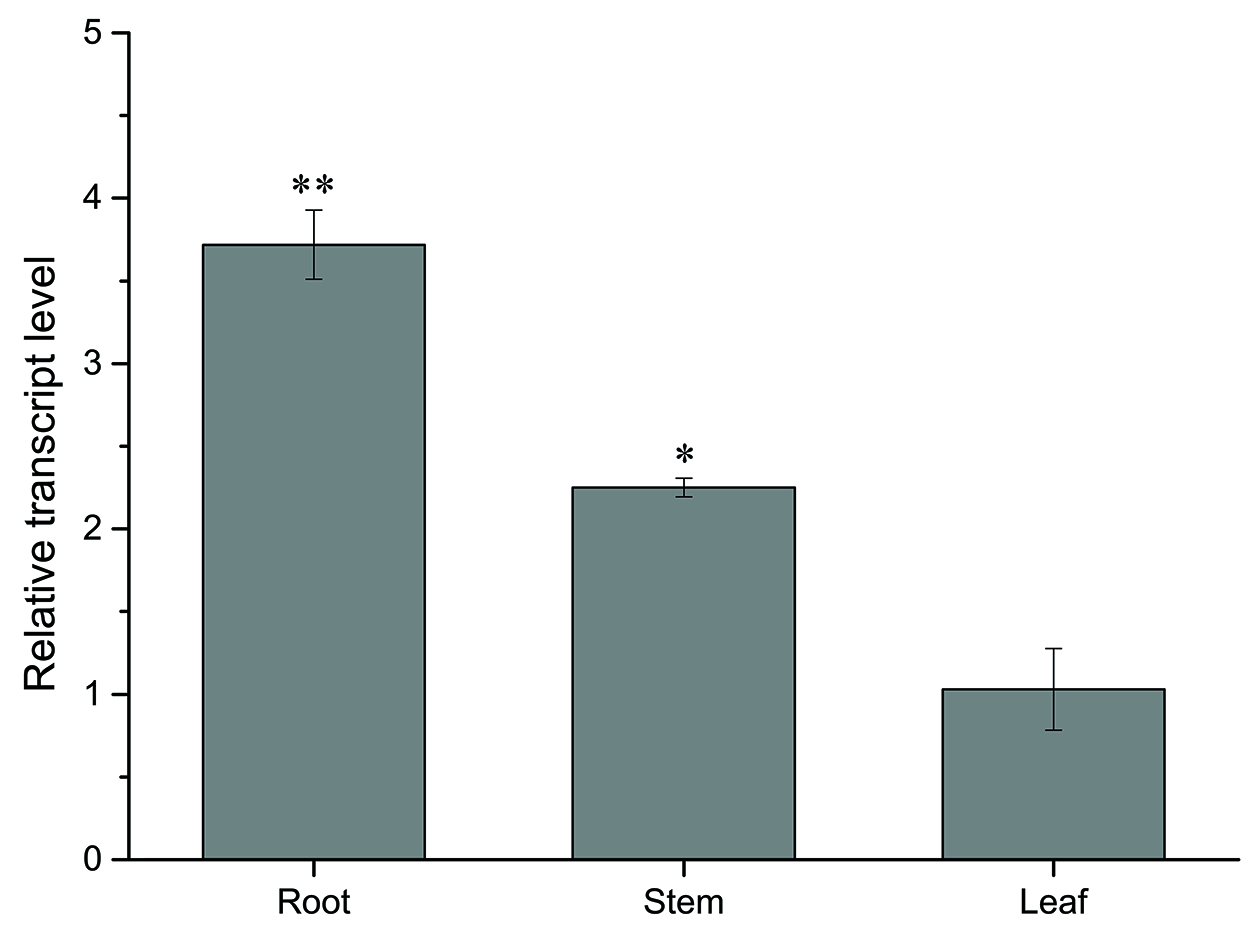

Supplement: S4 Fig — Data were collected from three independent biological repeats. Results are shown as means ±SE (n = 3). Asterisks indicate significant differences (* p<0.05, **p<0.01). (TIF) [file pone.0178101.s004.tif]

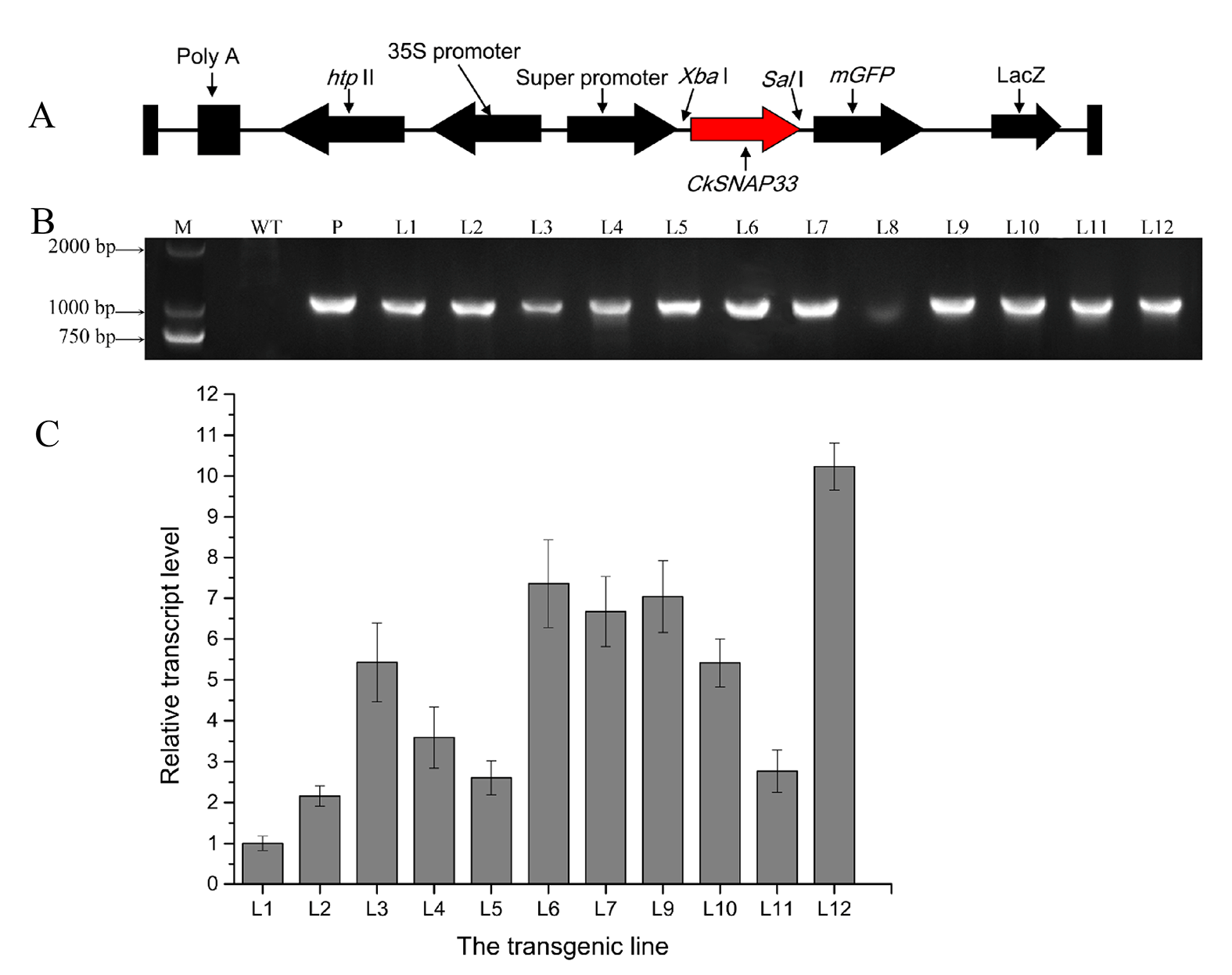

Supplement: S5 Fig — (A) Schematic representation of the modified pCAMBIA 1300 vector with CkSNAP33 under the control of a super promoter. (B) Genomic DNA-PCR analysis of genomic DNA from hygromycin B resistant lines. (C) Real-time PCR confirmed the expression of CkSNAP33 in transgenic lines. (TIF) [file pone.0178101.s005.tif]
