# Supplementary material for: Identification of CkSNAP33, a gene encoding synaptosomal-associated protein from Cynanchum komarovii, that enhances Arabidopsis resistance to Verticillium dahliae
Source: PLoS One. 2017 Jun 2;12(6):e0178101. doi: 10.1371/journal.pone.0178101 (PMC5456056; doi:10.1371/journal.pone.0178101)
Supplement: S1 Table — (DOCX) [file pone.0178101.s006.docx]

**S1 Table. List of primers used in this study**

| Primer | Sequence (5'→3') |
| --- | --- |
| qEF1α-F | TCAGGAAGCTCTTCCTGGTG |
| qEF1α-R | CAATGTGAGAGGTGTGGCAG |
| q33-F | AGCAGCCAATGGCTGTCTGA |
| q33-R | GGAGAAGAGACCACCAAGAC |
| ZW33-F | AGGCTCTAGAATGCTTGGCCTTAAGAAATC |
| ZW33-R | AAGCGTCGACTCTTTCCAAGTAAACGGCGT |
| 1300-F | GCCATTTCGCCTTTTCAG |
| 1300-R | GCTGAACTTGTGGCCGTT |
| qAtEF1α-F | CCTGGATTGCCACACC |
| qAtEF1α-F | AGTCTGCCTCATGTCC |
| qVd-F | AAAGTTTTAATGGTTCGCTAAGA |
| qVd-R | CTTGGTCATTTAGAGGAAGTAA |
| qAtPR1-F | CATACACTCTGGTGGGCCTT |
| qAtPR1-R | CTCACTTTGGCACATCCGAG |
| qAtPR5-F | TCACTCTAGTAGGCGATGGCG |
| qAtPR5-R | GCAGGCCACGACATTGTTCTG |

The enzyme restriction sites were underlined.
